# Collective Narcissism and In-Group Satisfaction Are Associated With Different Emotional Profiles and Psychological Wellbeing

**DOI:** 10.3389/fpsyg.2019.00203

**Published:** 2019-02-14

**Authors:** Agnieszka Golec de Zavala

**Affiliations:** ^1^Department of Psychology, Goldsmiths, University of London, London, United Kingdom; ^2^Department of Psychology, SWPS University of Social Sciences and Humanities, Poznań, Poland; ^3^ISCTE – Instituto Universitário de Lisboa, Lisbon, Portugal

**Keywords:** collective narcissism, in-group satisfaction, emotionality, pro-sociality, life satisfaction

## Abstract

The social identity approach to wellbeing posits that social identifications provide psychological resources that contribute to individual wellbeing. Unless individuals identify with stigmatized groups or groups whose norms prescribe damaging behaviors, identifying with groups seems beneficial. This article explores the possibility that the different ways individuals approach *the same* social identity (labeled collective narcissism vs. in-group satisfaction) may be differentially associated with wellbeing. Results of four studies indicate that collective narcissism (a belief that the in-group’s exceptionality is not sufficiently appreciated by others) vs. in-group satisfaction, (a belief that the in-group is of a high value), although positively correlated, are associated with different emotional profiles. In Study 1A (*N* = 570, in Poland) and Study 1B (*N* = 778, in the United States), collective narcissism was uniquely positively associated with negative emotionality, whereas in-group satisfaction was positively associated with positive emotionality and negatively associated with negative emotionality. In Study 2 (*N* = 569, in Poland), collective narcissism and in-group satisfaction had opposite unique links with social connectedness, gratitude and self-criticism. In Study 3 (*N* = 393, in Poland), collective narcissism, but not in-group satisfaction, was associated with sensory processing sensitivity, genetically determined hypersensitivity to negative stimuli. Collective narcissism was associated with life satisfaction only via its link to in-group satisfaction. Together these results suggest that dispositional negative emotionality may incline individuals toward collective narcissism. The positive overlap with in-group satisfaction may link collective narcissism to the benefits of social identification and wellbeing.

## Introduction

This article seeks to reconcile the seemingly contradictory findings that people who feel positive about themselves and others (e.g., Gramzow and Gaertner, 2005; Amiot and Aubin, 2013) and people who do not (Gusfield, 1963; Hofstadter, 1965/2008; Fromm, 1973; Lipset and Raab, 1973; Adorno, 1997; Golec de Zavala et al., 2019b), claim their group (the in-group) is of a high value. Previous studies showed that positive social identifications provide psychological resources (e.g., clear self-definition, higher self-esteem, a sense of meaning and direction, a sense of social connectedness) that support individual wellbeing. However, this does not apply to identifications with groups that are stigmatized or groups whose norms prescribe behaviors detrimental to wellbeing (for review see, Cruwys et al., 2014). Thus, the positive impact of sharing a social identity depends on its normative content and its intergroup status (Jetten et al., 2014). Going beyond previous studies, this article explores the possibility that the different ways *the same* social identity is perceived – which we label collective narcissism vs. in-group satisfaction – may be differentially linked to factors associated with wellbeing.

Collective narcissism and in-group satisfaction pertain to positive beliefs people may hold about the status and value of the social identity they share. Collective narcissism is a belief that the in-group is exceptional, entitled to privileged treatment but not sufficiently recognized by others (Golec de Zavala, 2018; Golec de Zavala et al., 2019a)^1^. In-group satisfaction is a belief that the in-group and one’s membership in it are the reasons to be proud of Leach et al. (2008). This article advances the idea that collective narcissism is uniquely associated with factors indicating low psychological wellbeing: negative emotionality, lack of life satisfaction (Diener et al., 1985) and social connectedness, and the inability to experience self-transcendent emotions that link people to someone or something beyond themselves (Stellar et al., 2017), such as gratitude (appreciating positive aspects of experience, feeling thankful to something or someone, Fredrickson, 2013) or compassion (sympathizing with suffering of others and a wish to relieve it, Gilbert, 2010). Thus, sharing a social identity may not offer psychological resources supporting wellbeing when individuals hold a collective narcissist belief about the in-group. Indeed, it is argued that dispositional negative emotionality inclines individuals toward collective narcissism. Conversely, in-group satisfaction is uniquely associated with factors indicating high wellbeing: positive emotionality, pro-sociality, and life satisfaction. Due to the positive overlap between in-group satisfaction and collective narcissism, the link between collective narcissism and negative emotionality may be reduced and collective narcissism may be *indirectly* linked to positive emotionality, pro-sociality and life satisfaction.

### Collective Narcissism and In-Group Satisfaction Have Opposite, Unique Relationships With Intergroup Hostility

Collective narcissism and in-group satisfaction pertain to ways people attribute value to their social identity. They positively overlap (for review see Golec de Zavala et al., 2019a), and differ from other aspects of social identity, such as in-group commitment or self-definition (Ellemers et al., 1999; Leach et al., 2008; Jaworska, 2016). Their positive correlations range from 0.31 to 0.63 (Golec de Zavala et al., 2019a). However, when their positive overlap is partialled out, collective narcissism and in-group satisfaction have opposite, unique relationships with intergroup hostility (Golec de Zavala et al., 2013a) and self-esteem (Golec de Zavala et al., 2019b).

Research shows that collective narcissism, but not in-group satisfaction, predicts intergroup hostility (for review see, Golec de Zavala et al., 2019a). Collective narcissism and in-group satisfaction have opposite associations with hostile attribution bias. Collective narcissism is related to intergroup distrust and a tendency to perceive out-groups as hostile toward the in-group, while in-group satisfaction is associated with a tendency to perceive out-groups as trustworthy and benevolent (Golec de Zavala et al., 2019a). Collective narcissism, but not in-group satisfaction, is associated with a tendency to believe in conspiracy theories about out-groups’ antagonistic intentions and plotting against the in-group (Golec de Zavala and Cichocka, 2012; Cichocka et al., 2016; Golec de Zavala and Federico, 2018). Collective narcissism, but not in-group satisfaction, predicts hypersensitivity and hostile retaliation to intergroup threat (Golec de Zavala et al., 2013b, 2016). Importantly, the link between collective narcissism and intergroup hostility is suppressed to the extent collective narcissism overlaps with in-group satisfaction (Golec de Zavala et al., 2013a). Such findings suggest that different associations with intergroup hostility may reflect a more basic difference in emotionality associated with collective narcissism and in-group satisfaction. In addition, the association with in-group satisfaction may weaken the association of collective narcissism with negative emotionality.

### Collective Narcissism and In-Group Satisfaction Have Different Relationships With Self-Esteem and Individual Narcissism

The distinct associations between collective narcissism vs. in-group satisfaction and intergroup hostility may be related to the fact that the two beliefs about the social identity seem to reflect different motivations to identify with a valuable in-group. Research suggests that collective narcissism may be associated with a tendency to exaggerate the in-group’s importance to compensate for low self-esteem and to satisfy frustrated self-entitlement. On the other hand, in-group satisfaction seems to be associated with the need to apply positive aspects of the self to enhance the in-group.

In this vein, studies suggest that collective narcissism vs. in-group satisfaction have opposite, unique relationships with self-esteem (the belief that one is of a high value, Golec de Zavala et al., 2019b) and personal control (the belief in one’s ability to influence the course of one’s own life, Cichocka et al., 2017). In addition, studies indicate that collective narcissism is uniquely related to vulnerable individual narcissism – antagonistic self-entitlement manifesting in a distrustful and neurotic interpersonal style (Miller et al., 2017). A meta-analytical summary indicated that collective narcissism was associated with vulnerable narcissism in all studies, in which this aspect of individual narcissism was assessed. Across different countries, collective narcissism was associated with low self-esteem via vulnerable narcissism (Golec de Zavala, 2018). Thus, the belief that the in-group’s exceptionality is not sufficiently recognized by others is associated with a similar belief about the self. Moreover, such a belief about the self seems to motivate collective narcissism (Golec de Zavala et al., 2019b).

Conversely, studies show that people whose self-esteem is high tend to project their positive self-evaluation onto their social identities (Gramzow and Gaertner, 2005; van Veelen et al., 2011). In addition, the positive belief about the in-group, which we label in-group satisfaction, is associated with the belief that positive characteristics of individuals should be used to enhance the valuable in-group (Amiot and Sansfaçon, 2011; Jans et al., 2011; Legault and Amiot, 2014). Thus, in-group satisfaction seems to be associated with positive and prosocial emotionality. Moreover, this association is reciprocal. For example, longitudinal studies showed that high self-esteem and sense of personal control measured in Time 1 increased in-group satisfaction assessed in Time 2. However, in-group satisfaction measured in Time 1 also increased self-esteem and the sense of personal control measured in Time 2 (Cichocka et al., 2017; Golec de Zavala et al., 2019b). Importantly, previous studies showed that in-group satisfaction suppressed the negative link between collective narcissism and self-esteem (Golec de Zavala et al., 2019b). Thus, the overlap with in-group satisfaction may offer a possibility to change negative emotionality associated with collective narcissism.

### Collective Narcissism, In-Group Satisfaction, and Emotionality

According to the two factor theory of affect (Diener and Larsen, 1984; Emmons and Diener, 1985), negative affect has a dispositional etiology, whereas positive emotionality is shaped by environmental experiences. Indeed, research suggests that individual differences in negative emotionality are genetically driven, whereas individual differences in positive emotionality are environmentally driven (Baker et al., 1992; Tackett et al., 2013; Zheng et al., 2016). Thus, the present studies explore the proposition that dispositional negative emotionality may incline people toward collective narcissism. However, by the virtue of the overlap between collective narcissism and in-group satisfaction, the negative emotionality can be changed toward greater positivity.

This proposition is supported by previous research indicating different unique associations of collective narcissism and in-group satisfaction with self-evaluation and intergroup attitudes. The present research builds also on previous studies indicating that in-group satisfaction suppresses the relationship between collective narcissism and a low sense of personal control (Cichocka et al., 2017), low self-esteem (Golec de Zavala et al., 2019b) and out-group derogation (Golec de Zavala et al., 2013a). The present research advances the proposition that in-group satisfaction may suppress the associations of collective narcissism with negative emotionality and it may drive the indirect positive associations between collective narcissism and positive and prosocial emotionality and life satisfaction. Although emotionality is a relatively stable way of relating to the outside world (Lee and Robbins, 2000), sustainable change over time is possible (Williams and Galliher, 2006). Experiencing positive emotions builds enduring physical, cognitive and social resources that help the recovery from negative emotions. Following the logic of an upward spiral, positive emotions produce more positive emotions and strengthen the ability to effectively alleviate the effects of negative emotions and maintain life satisfaction, even during hardship and adversity (Fredrickson, 2001). Capitalizing on the overlap with in-group satisfaction may be a way of reducing collective narcissistic hypersensitivity to negative and threatening stimuli and emotional negativity. Such a process may be reflected by the proposed indirect positive associations between collective narcissism and positive emotionality, pro-sociality and life satisfaction via in-group satisfaction.

### Overview

This article posits that collective narcissism is uniquely positively associated with negative emotionality and uniquely negatively associated with self-transcendent emotions and social connectedness. On the other hand, in-group satisfaction is uniquely associated with positive and prosocial emotionality, social connectedness and life satisfaction (Hypothesis 1). In addition, the present studies test the proposition that as long as collective narcissism overlaps with in-group satisfaction, not only might the association with negative emotionality weaken, but also collective narcissism may be indirectly associated with positive emotionality, pro-sociality, and life satisfaction via in-group satisfaction (Hypothesis 2).

Studies 1A and 1B examine the associations between collective narcissism vs. in-group satisfaction and positive and negative emotionality in Poland and in the United States to explore whether the tested associations can be found in different countries with different cultural norms governing emotional expressions. Cultural norms prescribe what emotions should be experienced and expressed, and how, and which emotions should be regulated (Markus and Kitayama, 1991). For example, it is normative in the United States to maximize positive emotions (Kitayama et al., 2000), whereas in Poland the cultural norm prescribes emotional frankness (Wierzbicka, 1999) and expression of negative emotionality (Wojciszke, 2004). Thus, the cultural norms may affect the expression of various emotions. Nevertheless, we expected that the predicted pattern of the relationships should remain unaffected when positive and negative emotionality is assessed with context-adequate measurements. Study 2 examine the associations between collective narcissism vs. in-group satisfaction and prosocial emotionality. Specifically, Study 2 tests whether collective narcissism vs. in-group satisfaction have opposite, unique associations with social connectedness and self-transcendent emotions such as gratitude and compassion.

In order to test the prediction that negative emotionality may predispose people toward collective narcissism, Study 3 explored the idea that collective narcissism might be associated with genetically based hypersensitivity to external (especially negative) stimuli (i.e., sensory processing sensitivity, Aron and Aron, 1997) (Hypothesis 3). Study 3 also tested the prediction that, despite this association in-group satisfaction would still drive the indirect positive link between collective narcissism and life satisfaction and that sensory processing sensitivity may be indirectly, positively linked to life satisfaction via collective narcissism and in-group satisfaction.

All the present studies are correlational. They do not make claims about directionality of tested associations with the exception of Study 3, where the genetically determined sensory processing sensitivity (a genetically based disposition) is expected to predict collective narcissism (a belief about the in-group). The remaining studies primarily explore the proposition that the associations of collective narcissism vs. in-group satisfaction with predictors of wellbeing may have different signs. Since all present studies are correlational, the term ‘indirect effect’ does not imply a causal effect (MacKinnon et al., 2007; Fiedler et al., 2011). Instead, this term is used to indicate a significant change in the relation between two variables when additional variables are statistically controlled for. Specifically, suppression occurs when one variable increases predictive validity of another variable and when a direct and indirect (via suppressor) relationship between two variables have opposite signs. Mediation occurs when one variable carries out the predictive validity of another variable and when a direct and an indirect (via mediator) relationship between two variables have the same signs (MacKinnon et al., 2000).

In all studies, a stepwise analytic strategy was applied to determine the unique associations of in-group satisfaction and collective narcissism with indices of positive, negative, and prosocial emotionality and life satisfaction. The studies tested whether correlations with those variables differed significantly for in-group satisfaction and collective narcissism (using Fisher’s *z*-test, Steiger, 1980). In order to assess unique associations of collective narcissism and in-group satisfaction with those variables, a series of partial correlations was also performed. Given that in-group satisfaction and collective narcissism are positively correlated, dominance weights were computed to assess the unique contribution of collective narcissism and in-group satisfaction explaining the variance in emotionality and life satisfaction. Regression weights of strongly correlated predictors alone may not give an adequate indicator of the unique contribution of each variable because they change with covariance relationships. Therefore, they may be sample-specific and not easily generalizable. Dominance weights give a more accurate assessment of the hierarchy of importance of the correlated predictors (Braun and Oswald, 2011). Finally, the hypothesized indirect effects were analyzed using multiple regression analyses.

Three studies were conducted in Poland, where the collective narcissistic rhetoric about the country’s threatened and misunderstood greatness has been increasingly present in public life, especially since the ultra-conservative, populist party *Prawo i Sprawiedliwosc* (*Law and Justice*) came to power (Hedges, 2017). Study 1B was conducted in the United States Similar to Poland, collective narcissism has been mobilized by the populist president Trump campaign and was associated with his electoral success (Federico and Golec de Zavala, 2018).

## Study 1A

Studies 1A and 1B examined the associations between collective narcissism vs. in-group satisfaction and positive and negative emotionality. They tested the hypothesis that collective narcissism would be uniquely associated with frequently experiencing negative emotions, whereas in-group satisfaction would be uniquely associated with frequently experiencing positive emotions (Hypothesis 1). In addition, they tested the hypothesis that in-group satisfaction would suppress the positive relationship between collective narcissism and negative emotionality and collective narcissism should be indirectly associated with positive emotionality via in-group satisfaction (Hypothesis 2). Study 1A was conducted in Poland, whereas Study 1B was conducted in the United States.

### Methods

#### Participants and Procedure

Study 1A was conducted among 570 Polish adults (294 female, *M*_Age_ = 44.11 years, *SD*_Age_ = 15.13). All datasets can be found in Supplementary Materials. Data collection was supported by the Ariadna Research Panel^2^. In order to estimate the required sample size, David Kenny’s MedPower app was used^3^. The effect sizes from previous studies were used: for the link between collective narcissism and in-group satisfaction, *r*_a_ = 0.31, (Golec de Zavala et al., 2016), and in-group satisfaction and positive emotionality, *r*_b_ = 0.32; and in-group satisfaction and negative emotionality, *r*_b_ = -0.35 (Yampolsky and Amiot, 2013). The links between collective narcissism and positive and negative emotionality were conservatively assumed to be small, *r*_c_ = 0.10. The most conservative estimation indicated a minimum sample size of 102 participants to detect the hypothesized indirect links with 80% of statistical power. The study conservatively oversampled following the suggestion that the size of correlations stabilizes in samples larger than *N* = 250 (Schönbrodt and Perugini, 2013). The data collection ceased on a predetermined date. After giving their consent, participants first responded to demographic questions. Next, they received the measures and items within measures in a separate random order for each participant.

#### Measures

*Collective narcissism* was measured by the 5-item Collective Narcissism Scale used in previous studies (Golec de Zavala et al., 2009, 2013a). Items include statements such as: “I will not be satisfied until the Polish nation obtains respect it deserves.” Participants indicated their responses on a scale of 1 (*completely disagree*) to 7 (*completely agree*), α = 0.90, *M* = 4.41; *SD* = 1.35.

*In-group satisfaction* was assessed by the in-group satisfaction subscale of the In-group Identity Scale (Leach et al., 2008) as in previous studies (Golec de Zavala et al., 2013a, 2016, 2019b). Items include statements such as: “I am glad to be a member of my national group.” Participants indicated their responses on a scale of 1 (*completely disagree*) to 7 (*completely agree*), α = 0.92; *M* = 5.08; *SD* = 1.36.

*Positive and negative emotionality* were measured using 11 items corresponding to the 11 subscales of the Positive and Negative Affect Scale X (Watson and Clark, 1999) from a well-validated Polish adaptation (Fajkowska and Marszał-Wiśniewska, 2009). The extended version of PANAS scale assesses specific qualities of positive and negative affect. In the present study, participants were asked which of the following emotions they experienced: joy, alertness, confidence, calm (positive), sadness, fear, guilt, hostility (negative), calm, shyness, tiredness, upset (other). Participants responded using a scale ranging from 1 (*not at all*) to 7 (*to a large degree*). Based on the results of the Principal Components Factor analysis (see below), two scales were computed corresponding to positive (joy, alertness, confidence, calm, α = 0.75; *M* = 4.56; *SD* = 0.98) and negative emotionality (sadness, fear, guilt, hostility, shyness, tiredness, upset, α = 0.86; *M* = 3.13; *SD* = 1.07).

### Results and Discussion

#### Preliminary Analyses

A Principal Components Factor Analysis performed for the items on the Collective Narcissism Scale and the In-group Satisfaction Scale revealed a two-factor solution, with factor loadings larger than 1 together explaining 74.90% of variance. Collective narcissism items loaded on the first factor with factor loading 5.69 and item loadings greater than 0.70. In-group satisfaction items loaded on the second factor with factor loading 1.05 and item loadings greater than 0.80. A Principal Components Factor Analysis performed on the items corresponding to the PANAS-X scales revealed a two-factor solution, with factor loadings larger than 1 together explaining 57.87% of variance. Negative emotions loaded on the first factor with factor loading 4.22 and item loadings greater than 0.48. Positive emotions loaded on the second factor with factor loading 2.06 and item loadings greater than 0.70. The scales were negatively correlated, *r*(568) = -0.26, <0.001.

#### Collective Narcissism, In-Group Satisfaction and Positive and Negative Emotionality

Correlational analyses in Table 1 showed that collective narcissism and in-group satisfaction were positively correlated. Collective narcissism was positively correlated with positive and negative emotionality, whereas in-group satisfaction was positively correlated with positive emotionality and negatively correlated with negative emotionality.

**Table 1:** Associations between the variables in Study 1A (*N* = 570).

	Collective narcissism			In-group satisfaction			Fisher *z*
			
	*r*	Partial	Dominance	*r*	Partial	Dominance	
Positive emotionality	0.25^***^	-0.01	0.03	0.36^***^	0.27^***^	0.09	-3.53^***^
Negative emotionality	0.13^**^	0.28^***^	0.06	-0.10^*^	-0.27^***^	0.02	-7.02^***^
Intercorrelation	0.69^∗∗∗^						


*The intercorrelation refers to that between collective narcissism and in-group satisfaction; ^∗^*p* < 0.05, ^∗∗^*p* < 0.001, ^∗∗∗^*p* < 0.001.*

Comparisons of dependent correlations (comparing associations of collective narcissism to associations of in-group satisfaction) indicated that the correlation of in-group satisfaction with positive emotionality was significantly stronger than the correlation of collective narcissism with positive emotionality, and the correlation of collective narcissism with negative emotionality was stronger than the correlation of in-group satisfaction with negative emotionality (with opposite sign). Dominance weights suggested that collective narcissism explained more variance in negative emotionality, whereas in-group satisfaction explained more variance in positive emotionality.

Partial correlations clarified additionally that collective narcissism was uniquely, positively associated only with negative emotionality (but not with positive emotionality). In-group satisfaction was uniquely positively linked to positive emotionality and uniquely negatively associated with negative emotionality.

#### Indirect Relationships

The analyses of indirect relationships (using PROCESS macro for SPSS Model 4, Hayes, 2013, Table 2) indicated that collective narcissism was directly positively associated with negative emotionality. It was also indirectly, negatively associated with negative emotionality, via in-group satisfaction. Collective narcissism had no unique, direct association with positive emotionality. However, the indirect, positive link between collective narcissism and positive emotionality via in-group satisfaction was significant.

**Table 2:** Direct and indirect relationships of collective narcissism and in-group satisfaction with positive and negative emotionality, Study 1 (*N* = 570).

	Positive emotionality				Negative emotionality			
		
	Direct effect *b(SE)*	IE	95%CI	Sobel *z*	Direct effect *b(SE)*	IE	95%CI	Sobel *z*
CN → IS	-0.01(0.04)	0.18(0.03)	[0.13; 0.25]	6.48^***^	0.31(0.04)^∗∗∗^	-0.20(0.03)	[-0.27;-0.14]	-6.37^***^
IS → CN	0.26 (0.04)^∗∗∗^	-0.003(0.03)	[-0.06; 0.05]	-0.11	-0.29(0.04)^∗∗∗^	0.21(03)	[0.15; 0.28]	6.66^***^


**^∗∗∗^p < 0.001.**

In-group satisfaction was directly, positively associated with positive emotionality and this relationship was not affected by its overlap with collective narcissism. In-group satisfaction was directly, negatively associated with negative emotionality. It was also indirectly, positively associated with negative emotionality, via collective narcissism. This suppression explains a non-significant zero-order correlation between in-group satisfaction and negative emotionality.

In sum, the results of Study 1A support Hypothesis 1 indicating that collective narcissism is uniquely associated with negative emotionality, whereas in-group satisfaction is uniquely associated with positive emotionality. The results also support Hypothesis 2 indicating that because collective narcissism positively overlaps with in-group satisfaction, its association with negative emotionality is weakened. In addition, collective narcissism is indirectly associated with positive emotionality via in-group satisfaction. Moreover, the present results show that in-group satisfaction is not only uniquely positively associated with positive emotionality, but it is also uniquely, negatively associated with negative emotionality. It is positively associated with negative emotionality only via collective narcissism. The positive overlap between collective narcissism and in-group satisfaction does not suppress the positive link between in-group satisfaction and positive emotionality.

## Study 1B

Study 1B was conducted in the United States It closely followed the procedure used in Study 1A but used the measure of positive and negative emotionality relevant to this national context.

### Methods

#### Participants and Procedure

Study 1B was conducted among 778 American Mturk workers (374 female, *M*_Age_ = 36.33 years, *SD*_Age_ = 14.66)^4^. After giving their consent, participants first responded to demographic questions. Next, they received the measures and items within measures in a separate random order for each participant. Participants were paid a small fee in exchange for their participation. Only the data from participants who responded to all measures were analyzed.

#### Measures

*Collective narcissism*, α = 0.89, *M* = 3.95, *SD* = 1.47 and *In-group satisfaction* α = 0.94; *M* = 5.13, *SD* = 1.51 were with the same items measured as in Study 1A. Participants responded using a scale from 1 (*strongly agree*) to 7 (*strongly disagree*). The items were recoded so the higher score on the scales indicates the higher level of the variable.

*Positive and negative emotionality* were measured using 10 items of the International Positive and Negative Affect Scale Short Form (I-PANAS-SF, Thompson, 2007). In the present study, participants were asked to what extent they felt: alert, inspired, determined, attentive and active (positive emotionality) and upset, hostile, guilty, nervous and ashamed. Participants responded using the scale ranging from 1 (*not at all*) to 5 (*to a large degree*). Based on the results of the Principal Components Factor analysis described below, two scales were computed corresponding to negative (α = 0.93; *M* = 1.77; *SD* = 1.06) and positive emotionality (α = 0.81; *M* = 3.56; *SD* = 0.89).

### Results and Discussion

#### Preliminary Analyses

A Principal Components Factor Analysis performed for the items on the Collective Narcissism Scale and the In-group Satisfaction Scale revealed a two-factor solution, with factor loadings larger than 1 together explaining 76.80% of variance. In-group satisfaction items loaded on the first factor with factor loading 5.45 and item loadings greater than 0.86. Collective narcissism items loaded on the second factor with factor loading 1.45 and item loadings greater than 0.68. A Principal Components Factor Analysis performed on the items corresponding to the I-PANAS-SF scales revealed a two-factor solution, with factor loadings larger than 1 together explaining 67.95% of variance. Negative emotions loaded on the first factor with factor loading 3.93 and item loadings greater than 0.84. Positive emotions loaded on the second factor with factor loading 2.86 and item loadings greater than 0.72. The scales were uncorrelated, *r*(776) = -0.03, *p* = 0.47.

#### Collective Narcissism, In-Group Satisfaction and Positive and Negative Emotionality

Correlation analyses in Table 3 showed that collective narcissism and in-group satisfaction were positively correlated. Collective narcissism was positively correlated with positive and negative emotionality, whereas in-group satisfaction was positively correlated with positive emotionality and negatively correlated with negative emotionality.

**Table 3:** Associations between the variables in Study 1B (*N* = 778).

	Collective narcissism			In-group satisfaction			Fisher *z*
			
	*r*	Partial	Dominance	*r*	Partial	Dominance	
Positive emotionality	0.16^***^	0.06	0.01	0.19^***^	0.12^**^	0.03	-0.95
Negative emotionality	0.22^***^	0.42^***^	0.11	-0.19^***^	-0.41^***^	0.10	13.16^***^
Intercorrelation	0.60^∗∗∗^						


*The intercorrelation refers to that between collective narcissism and in-group satisfaction; ^∗∗^*p* < 0.01, ^∗∗∗^*p* < 0.001.*

Comparisons of dependent correlations indicated that the correlation of in-group satisfaction with positive emotionality was not significantly different from the correlation of collective narcissism and positive emotionality. Dominance weights suggest that the contribution of in-group satisfaction to explaining the variance in positive emotionality was slightly stronger than the contribution of collective narcissism. Comparisons of dependent correlations indicated that the correlation of in-group satisfaction with negative emotionality was significantly different from the correlation of collective narcissism with negative emotionality. Dominance weights suggested that collective narcissism and in-group satisfaction explained almost the same amount of variance in negative emotionality (with opposite signs).

Partial correlation clarified that, just like in Study 1A, collective narcissism did not have unique association with positive emotionality. Instead, it was uniquely, positively associated with negative emotionality. In-group satisfaction was, as in Study 1A, uniquely, positively associated with positive emotionality and uniquely, negatively associated with negative emotionality.

#### Indirect Relationships

The analyses of indirect relationships (using PROCESS macro for SPSS Model 4, Hayes, 2013, Table 4) indicated that collective narcissism was directly, positively linked to negative emotionality. It was also indirectly, negatively associated with negative emotionality, via in-group satisfaction. Collective narcissism had no unique, direct association with positive emotionality. However, the indirect, positive link between collective narcissism and positive emotionality via in-group satisfaction was significant.

**Table 4:** Direct and indirect associations of collective narcissism and in-group satisfaction with positive and negative emotionality, Study 1B (*N* = 778).

	Positive emotionality				Negative emotionality			
		
	Direct effect *b(SE)*	IE	95%CI	Sobel *z*	Direct effect *b(SE)*	IE	95%CI	Sobel *z*
CN → IS	0.04(0.03)	0.05(0.02)	[0.02; 0.09]	3.25^**^	0.38(0.03)^∗∗∗^	-0.22(0.03)	[-0.27; -0.17]	-10.80^***^
IS → CN	0.09 (0.03)^∗∗^	0.03(0.02)	[-0.01; 0.06]	1.56	-0.35(0.03)^∗∗∗^	0.22(02)	[0.18; 0.27]	11.02^***^


*^∗∗^ p < 0.01, ^∗∗∗^p < 0.001.*

In-group satisfaction was directly, positively associated with positive emotionality and its overlap with collective narcissism did not reduce this association significantly. In-group satisfaction was directly, negatively associated with negative emotionality. However, due to its overlap with collective narcissism, in-group satisfaction was also indirectly, positively associated with negative emotionality.

In sum, the findings are remarkably consistent across Studies 1A and 1B. They indicate that collective narcissism is uniquely associated with negative emotionality and in-group satisfaction is uniquely, positively associated with positive emotionality. Those results support Hypothesis 1. In addition, findings indicate that in-group satisfaction is also uniquely, negatively associated with negative emotionality. In line with Hypothesis 2, in both countries, the association between collective narcissism and negative emotionality was reduced due to the positive overlap between collective narcissism and in-group satisfaction. In addition, collective narcissism was indirectly linked to positive emotionality via in-group satisfaction. The results indicate additionally, that in-group satisfaction was indirectly related to negative emotionality via its association with collective narcissism.

## Study 2

Study 2 extended the examination of the associations of collective narcissism and in-group satisfaction into the domain of prosocial, self-transcendent emotions. Study 2 tested the prediction that in-group satisfaction would be uniquely, positively associated with social connectedness (self-assessed ability to form meaningful social relations, Lee et al., 2001), gratitude (e.g., Fredrickson, 2013), compassion (e.g., Gilbert, 2010), and self-compassion (a kind and tolerant attitude toward oneself, Neff, 2003). Study 2 tested the prediction that collective narcissism would be uniquely, negatively linked to those variables, while in-group satisfaction would be uniquely, positively associated with those variables (Hypothesis 1). In addition, Study 2 tested the prediction that the positive overlap with in-group satisfaction would weaken the negative associations of collective narcissism and compassion, self-compassion, social connectedness, and gratitude (Hypothesis 2).

### Methods

#### Participants and Procedure

Study 2 was conducted among 569 Polish adults (313 females, *M*_Age_ = 44.72 years, *SD*_Age_ = 15.70)^5^. Data collection was supported by the Ariadna Research Panel. Participants responded to an online survey allegedly assessing self-views and their perception of Poland. The sample size estimation followed the logic of that performed for Studies 1A and B. The data collection ceased on a predetermined date. All measures were presented in random order, and the order of the items was also randomized. Unless otherwise indicated all items were answered on a scale from 1 (*completely disagree*) to 6 (*completely agree*).

#### Measures

*Collective narcissism*, α = 0.90, *M* = 3.49, *SD* = 1.18 and *In-group satisfaction*, α = 0.96, *M* = 4.43, *SD* = 1.25 were measured as in previous studies.

Social connectedness was measured using the 8-item Revised Social Connectedness Scale (Lee and Robbins, 1995; Lee et al., 2001). Items included statements such as: “I feel disconnected from the world around me.” The scale was translated to Polish and back-translated by independent bilingual speakers. The items were recoded so the higher indicates higher social connectedness. The scale had high reliability, *α* = 0.86, *M* = 3.87, *SD* = 0.96.

*Gratitude* was measured using the 6-item Gratitude Questionnaire (McCullough et al., 2002). Items included statements such as: “I have so much in life to be thankful for.” The scale was translated to Polish and back-translated by independent bilingual speakers. The scale had high reliability, α = 0.81, *M* = 4.11, *SD* = 1.02.

*Compassion* was measured by the Santa Clara Brief Compassion Scale (Hwang et al., 2008). Items includes statements such as: “I tend to be compassionate for people even though I don’t know them.” The scale was translated to Polish and back-translated by independent bilingual speakers. Due to a clerical error only 4 out of 5 items were included in the study. The items formed a reliable scale, with reliability at *α* = 0.81, *M* = 4.16, *SD* = 0.90.

*Self-compassion and self-criticism* were measured by the 12-items Self-Compassion Scale (Neff, 2003; Raes et al., 2011). The scale was translated to Polish and back-translated by independent bilingual speakers. The Principal Components Factor analysis for this scale produced a two-factor solution with uncorrelated factors (see below). Thus, a self-compassion index, α = 0.79, *M* = 3.83, *SD* = 0.73 and a self-criticism index, α = 0.84, *M* = 3.47, *SD* = 0.90 were created. Items of the self-compassion index included statements such as: “When I feel inadequate in some way, I try to remind myself that feelings of inadequacy are shared by most people.” Items of the self-criticism index included statements such as: “I’m intolerant and impatient toward those aspects of my personality I don’t like.”

### Results and Discussion

#### Preliminary Analyses

A Principal Components Factor Analysis performed for the items on the Collective Narcissism Scale and the In-group Satisfaction Scale revealed a two-factor solution, with factor loadings larger than 1 together explaining 80.36% of variance. In-group satisfaction items loaded on the first factor with factor loading 5.51 and item loadings greater than 0.89. Collective narcissism items loaded on the second factor with factor loading 1.74 and item loadings greater than 0.72. A Principal Components Factor Analysis for the items measuring social connectedness produced a one-factor solution with the factor loading of 4.16 explaining 51.95% of variance. Factor loadings were above 0.55. A Principal Components Factor Analysis for the items on the gratitude measure produced a one-factor solution with the factor loading of 3.16 explaining 52.82% of variance. Factor loadings were above 0.53. A Principal Components Factor Analysis for the items measuring compassion produced a one-factor solution with the factor loading of 3.05 explaining 76.12% of variance. Factor loadings were above 0.79.

A Principal Components Factor Analysis performed for the items on the Self-Compassion Scale produced a two-factor solution. Both factors explained 55.74% of variance. The reversely coded items loaded on one factor, whereas the positively coded items loaded on another factor. Six positively coded items were retained and performed a Principal Components Factor Analysis was performed again. This analysis produced a one-factor solution with the factor loading of 2.93 explaining 48.83% of variance with factor loadings greater than 0.57. Next, the analysis was performed for the reversely items in original wording pertaining to self-criticism. This analysis produced a one-factor solution with the factor loading of 3.38 explaining 56.31% of variance with factor loadings greater than 0.67. The factors formed two uncorrelated scales, *r*(567) = 0.04, *p* = 0.30.

#### Collective Narcissism, In-Group Satisfaction and Pro-sociality

Correlational analyses in Table 5 showed that collective narcissism and in-group satisfaction were positively associated. Collective narcissism was positively correlated with gratitude, compassion, self-compassion, and self-criticism. In-group satisfaction was positively correlated with social connectedness, gratitude, compassion, and self-compassion.

**Table 5:** Associations between the variables in Study 2 (*N* = 569).

	Collective narcissism			In-group satisfaction			Fisher *z*
			
	*r*	Partial	Dominance	*r*	Partial	Dominance	
Social connectedness	0.04	-0.11^**^	0.005	0.25^***^	0.26^***^	0.04	-5.27^***^
Gratitude	0.10^*^	-0.09^*^	0.007	0.33^***^	0.33^***^	0.08	-5.88^***^
Compassion	0.25^***^	0.08	0.04	0.35^***^	0.26^***^	0.09	2.61^***^
Self-compassion	0.17^***^	0.08	0.02	0.21^***^	0.14^***^	0.03	-1.00
Self-criticism	0.15^***^	0.22^***^	0.03	-0.06	-0.17^***^	0.02	5.20^***^
Intercorrelation	0.53^∗∗∗^						


*The intercorrelation refers to that between collective narcissism and in-group satisfaction; ^∗^p < 0.05, ^∗∗^p < 0.01, ^∗∗∗^p < 0.001.*

Comparisons of dependent correlations indicated that the correlations of in-group satisfaction with social connectedness, gratitude, and compassion were significantly stronger than the corresponding correlations of collective narcissism, while the correlations with self-compassion did not differ significantly. Dominance analyses indicated that in-group satisfaction explained variance in social connectedness, gratitude and compassion to a larger extent than collective narcissism. The contribution of collective narcissism and in-group satisfaction was comparable in case of self-compassion and self-criticism (with the opposite sign).

Partial correlations clarified that in-group satisfaction was uniquely, positively related to social connectedness, gratitude, compassion and self-compassion and uniquely, negatively associated with self-criticism. Collective narcissism was uniquely, positively associated with self-criticism and uniquely, negatively associated with gratitude and social connectedness. It had no unique association with compassion or self-compassion.

#### Indirect Relationships

The analyses of indirect relationships (using PROCESS macro for SPSS, Model 4, Hayes, 2013, Table 6) indicated that collective narcissism was directly, negatively associated with social connectedness and gratitude and directly, positively associated with self-criticism. Collective narcissism was indirectly, positively associated with social connectedness and gratitude via in-group satisfaction. It was also indirectly, negatively associated with self-criticism via in-group satisfaction. Collective narcissism had no unique, direct relationship with compassion or self-compassion. However, it was positively, indirectly associated with both variables via in-group satisfaction.

**Table 6:** Direct and indirect associations of collective narcissism and in-group satisfaction with indices of pro-sociality, Study 2 (*N* = 569).

	Social connectedness				Gratitude					Compassion				Self-compassion					Self-criticism			
							
	Direct effect *b(SE)*	IE	95%CI	Sobel *z*	Direct effect *b(SE)*	IE	95%CI	Sobel *z*		Direct effect *b(SE)*	IE	95%CI	Sobel *z*	Direct effect *b(SE)*	IE	95%CI	Sobel *z*		Direct effect *b(SE)*	IE	95%CI	Sobel *z*
CN → IS	-0.10(0.04)^∗∗^	0.13(0.06)	[0.08; 0.19]	5.92^***^	-0.08(0.04)^∗^	0.15 (0.03)	[0.11; 0.21]	7.23^***^	CN → IS	0.07(0.04)	0.14(0.03)	[0.09; 0.20]	5.93^***^	0.05(0.03)	0.05(0.02)	[0.02; 0.09]	3.20^**^	CN → IS	0.19(0.04)^∗∗∗^	-0.08(0.02)	[-0.12; -0.03]	-3.84^***^
IS → CN	0.24(0.04)^∗∗∗^	-0.05(0.02)	[-0.10; -0.001]	-2.51^*^	0.28(0.03)^∗∗∗^	-0.04 (02)	[-0.08; 0.004]	-2.17^*^	IS → CN	0.24(0.04)^∗∗∗^	0.04(0.02)	[-0.01; 0.09]	1.89	0.09(0.03)^∗∗∗^	0.03(0.02)	[-0.01; 0.06]	1.81	IS → CN	-0.14(0.04)^∗∗∗^	0.10(02)	[0.05; 0.14]	4.94^***^


*^∗^p < 0.05, ^∗∗^p < 0.01, ^∗∗∗^p < 0.001.*

In-group satisfaction was directly, positively associated with compassion, self-compassion, social connectedness and gratitude, and directly, negatively associated with self-criticism. The positive overlap with collective narcissism did not affect the associations of in-group satisfaction with compassion and self-compassion. However, in-group satisfaction was indirectly, negatively related to social connectedness and gratitude via collective narcissism and indirectly, positively related to self-criticism via collective narcissism.

In sum, the results of Study 2 partially support Hypothesis 1, indicating that collective narcissism is uniquely, negatively associated with social connectedness and gratitude. The results indicating no unique, negative relationship between collective narcissism and compassion and self-compassion are not in line with Hypothesis 1. This may be due to the fact that out of the examined self-transcending emotions, compassion may be a subject to in-group bias and it is experienced with more ease and frequency toward in-group than out-group members (Stellar et al., 2017). Thus, there may be an incentive for collective narcissists to express in-group bias by selective compassion. In support of Hypothesis 1, the results also indicate that collective narcissism was uniquely, positively associated with self-criticism. The results indicating that in-group satisfaction is uniquely positively associated with social connectedness, gratitude, compassion and self-compassion, and uniquely negatively associated with self-criticism also support Hypothesis 1.

The results of Study 2 support Hypothesis 2 showing that due to the positive association between collective narcissism and in-group satisfaction, the negative relationships between collective narcissism and social connectedness, gratitude and self-criticism are weakened, and collective narcissism is indirectly linked to compassion and self-compassion via in-group satisfaction.

## Study 3

Study 3 analyzed the associations between collective narcissism vs. in-group satisfaction and life satisfaction. It tested the prediction that collective narcissism would be uniquely negatively related to life satisfaction, whereas in-group satisfaction would be uniquely positively related to life satisfaction (Hypothesis 1). It tested the prediction that collective narcissism would be associated with high life satisfaction only indirectly, via in-group satisfaction (Hypothesis 2). In addition, Study 3 sought to explore the expectation that collective narcissism might be associated with dispositional negative emotionality. It tested the prediction that collective narcissism, but not in-group satisfaction, was uniquely positively linked to sensory processing sensitivity (Hypothesis 3). Sensory processing sensitivity is a genetically determined elevated responsiveness to environmental stimuli involving deeper cognitive and sensory processing, including exaggerated experience of pain (Aron and Aron, 1997) and exaggerated responsiveness to negative stimuli (Jagiellowicz et al., 2011).

### Methods

#### Participants and Procedure

Study 3 was conducted among 393 Polish undergraduate students (287 females, 3 preferred not to disclose gender, *M*_Age_ = 26.65 years, *SD*_Age_ = 0.28) who participated in exchange for the course credit. The sample size estimation followed the logic of that performed for Studies 1–2. The data collection ceased on a predetermined date.

Data were collected as a part of a course assignment. Participants responded to an online survey allegedly assessing self-views and opinions about Poland. All measures were presented in random order, and the order of the items was randomized. Unless otherwise indicated all items were answered on a scale from 1 (*completely disagree*) to 6 (*completely agree*).

#### Measures

*Collective narcissism*, α = 0.86, *M* = 4.23, *SD* = 1.06 and *In-group satisfaction*, α = 0.93; *M* = 4.27, *SD* = 1.08 were measured as in previous studies.

*Life satisfaction* was measured with the Polish version of the 5-item Satisfaction with Life Scale (Diener et al., 1985; Jankowski, 2012). Items include statements such as: “In most ways my life is close to my ideal.” The scale had high reliability, α = 0.87, *M* = 3.53, *SD* = 1.05.

*Sensory processing sensitivity* was measured by the Polish version of the 27-item Highly Sensitive Person Scale (Aron and Aron, 1997) assessing individual differences in detecting and responding to sensory stimuli. Items of the scale pertain to responding to subtle environmental stimuli, being bothered by strong stimuli and getting easily startled and responding emotionally by external stimuli. Items include statements such as: “Are you easily overwhelmed by strong sensory input?” The scale was translated to Polish and back-translated by independent bilingual speakers. Responses were provided on a scale from 1 (*not at all*) to 6 (*very much so*), α = 0.88, *M* = 3.70, *SD* = 0.68.

### Results and Discussion

#### Preliminary Analyses

A Principal Components Factor Analysis performed for the items on the Collective Narcissism Scale and the In-group Satisfaction Scale revealed a two-factor solution, with factor loadings larger than 1 together explaining 73.25% of variance. In-group satisfaction items loaded on the first factor with factor loading 5.08 and item loadings greater than 0.80. Collective narcissism items loaded on the second factor with factor loading 1.52 and item loadings greater than 0.67. A Principal Components Factor Analysis for the items on the Life Satisfaction Scale produced a one-factor solution with the factor loading of 3.34 explaining 66.76% of variance. Factor loadings were above 0.74. A Principal Components Factor Analysis for the items on the Sensory Processing Sensitivity Scale revealed a three-factor solution corresponding to positively correlated Aesthetic Sensitivity, Low Sensory Threshold, and Ease of Excitation, factors differentiated by previous studies (Smolewska et al., 2006). All factors explained 40.61% of variance. The Low Sensory Threshold items loaded on the first factor with factor loading of 7.22. The Aesthetic Sensitivity items loaded on the second factor with factor loading of 2.04 and the Ease of Excitation items loaded on the third factor with factor loading of 1.70. The factors were positively correlated.

#### Collective Narcissism, In-Group Satisfaction and Life Satisfaction

Correlational analyses in Table 7 showed that collective narcissism and in-group satisfaction were positively correlated. Collective narcissism was positively correlated with sensory processing sensitivity and life satisfaction, whereas in-group satisfaction was positively correlated only with life satisfaction. Sensory processing sensitivity was negatively correlated with life satisfaction, *r*(931) = -0.19, *p* < 0.001.

**Table 7:** Associations between the variables in Study 3 (*N* = 939).

	Collective narcissism			In-group satisfaction			Fisher *z*
			
	*r*	Partial	Dominance	*r*	Partial	Dominance	
Life satisfaction	0.13^**^	0.006	0.01	0.22^***^	0.18^***^	0.04	-2.96^**^
Sensory processing sensitivity	0.14^**^	0.12^***^	0.02	0.08	-0.004	0.005	1.95^*^
Intercorrelation	0.55^∗∗∗^						


*The intercorrelation refers to that between collective narcissism and in-group satisfaction; ^∗^*p* = 0.051, ^∗∗^*p* < 0.01, ^∗∗∗^*p* < 0.001.*

Comparisons of dependent correlations indicated that the correlation of in-group satisfaction with life satisfaction was significantly stronger than the correlation of collective narcissism. The correlation of collective narcissism and sensory processing sensitivity was marginally stronger that the association of in-group satisfaction with this variable. Dominance weights suggested that in-group satisfaction explained variance in life satisfaction to a larger extent than collective narcissism and collective narcissism had a stronger association with sensory processing sensitivity than in-group satisfaction.

Partial correlation clarified that only in-group satisfaction was uniquely, positively linked to life satisfaction. It has no unique association with sensory processing sensitivity. Collective narcissism was uniquely, positively associated with sensory processing sensitivity. It had no unique relationship with life satisfaction.

#### Indirect Relationships

The analyses of indirect relationships (using PROCESS macro for SPSS, Model 4, Hayes, 2013, Table 8) indicated that collective narcissism had no direct association with life-satisfaction. Its association with life satisfaction was indirect and positive via in-group satisfaction. In-group satisfaction was directly, positively linked to life satisfaction. This link was not weakened by the positive overlap between in-group satisfaction and collective narcissism.

**Table 8:** Indirect and direct associations of collective narcissism and in-group satisfaction with life satisfaction, Study 3 (*N* = 393).

	Life satisfaction			
	
	Direct effect *b(SE)*	IE	95%CI	Sobel *z*
CN → IS	0.007 (0.06)	0.12 (0.04)	[0.06; 0.18]	3.56^***^
IS → CN	0.21 (0.06)^∗∗^	0.004 (0.03)	[-0.06; 0.07]	0.11


*^∗∗^p < 0.01; ^∗∗∗^p < 0.001.*

Those results support Hypothesis 1 indicating that collective narcissism does not have any unique association with life satisfaction. They support Hypothesis 2 indicating that collective narcissism is related to high life satisfaction only via in-group satisfaction. Finally, the results also support Hypothesis 3 that emotional negativity associated with collective narcissism may be dispositional. They indicate that collective narcissism is positively associated with sensory processing sensitivity, a factor contributing to higher anxiety and depression (Liss et al., 2008; Bakker and Moulding, 2012).

**FIGURE 1:**
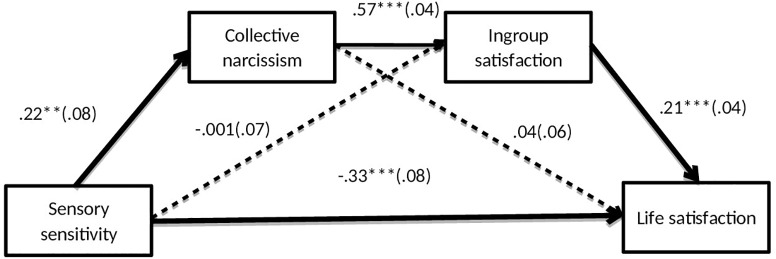
Relations among Variables in Study 3 (*N* = 393). The entries are unstandardized regression coefficients. Standard errors are in parentheses, *F*(3,388) = 14.80, *R*^2^ = 10, *p* < 0.001; ^∗∗^*p* < 0.01; ^∗∗∗^*p* < 0.001.

The present results also suggest that sensory processing sensitivity may be positively, although indirectly, associated with life satisfaction. Serial multiple mediation analysis was performed to test this prediction entering sensory processing sensitivity as a predictor, life satisfaction as the outcome and collective narcissism and in-group satisfaction as serial mediators (using PROCESS macro for SPSS, Model 6, Hayes, 2013). The analysis produced a significant, negative, direct effect of sensory processing sensitivity on life satisfaction. It also produced a positive, indirect effect via collective narcissism and in-group satisfaction, *IE* = 0.03, *SE* = 0.01; 95%*CI* [-0.006; -0.06]. The indirect effect via collective narcissism, *IE* = 0.009, *SE* = 0.01; 95%*CI* [-0.01; 0.04] or via in-group satisfaction, *IE* = -0.001, *SE* = 0.02; 95%*CI* [-0.03; 0.03] were not significant (Figure 1). This suggests that, because of the positive overlap between collective narcissism and in-group satisfaction, a sensitive person can access the psychological resources offered by positive social identification to improve individual wellbeing.

## General Discussion

People who score high on the Collective Narcissism Scale agree that their in-group’s importance is not sufficiently recognized by others, their in-group deserves special treatment and they insist that their in-group must obtain special recognition (Golec de Zavala et al., 2009). People who score high on the In-group Satisfaction subscale agree that it feels good to be a member of their in-group and there are a lot of reasons to feel proud to be a member of the in-group (Leach et al., 2008). Although both variables pertain to in-group positivity, only collective narcissism is uniquely related to hypersensitivity to intergroup threat and intergroup hostility, whereas in-group satisfaction suppresses those links (Golec de Zavala et al., 2019a). Moreover, collective narcissism in uniquely related to low self-esteem and low personal control, while in-group satisfaction is uniquely related to high self-esteem and high personal control (Cichocka et al., 2017; Golec de Zavala et al., 2019b). In this paper, we posit that such distinct unique associations of otherwise positively correlated variables reflect distinct emotional profiles associated with collective narcissism vs. in-group satisfaction.

The results of the present studies converged to support this proposition. Collective narcissism is uniquely related to negative emotionality, negatively related to social connectedness and gratitude, unrelated to compassion and self-compassion and life satisfaction, but related to self-criticism. On the other hand, in-group satisfaction is uniquely associated with positive emotionality and negatively associated with negative emotionality. It is also uniquely associated with self-transcendent emotions, self-compassion and life-satisfaction, and negatively associated with self-criticism. Its overlap with collective narcissism does not weaken its positive association with positive emotionality, compassion or life satisfaction. Such results corroborate previous findings, linking in-group satisfaction to positive emotionality and psychological wellbeing (Amiot and Aubin, 2013; Yampolsky and Amiot, 2013). They also support the predictions of the social identity approach (Jetten et al., 2014) showing that positive evaluation of one’s own social identity is related to individual wellbeing.

At the same time, the present results qualify the predictions of the social identity approach. It has been suggested that the positive impact of sharing a social identity depends on its normative content and its intergroup status (Jetten et al., 2014). The present results suggest that social identification may be detrimental to individual wellbeing not only when the in-group is stigmatized or prescribes unhealthy behaviors. Social identification may be also detrimental to wellbeing when the positive evaluation of the in-group takes the form of collective narcissism. Although collective narcissism and in-group satisfaction pertain to the ways individual evaluate the same social identity, collective narcissism is systematically, uniquely linked to variables detrimental to wellbeing such as negative emotionality, self-criticism, low social connectedness or lack of gratitude. Unlike in-group satisfaction, collective narcissism does not have a unique, positive association with life satisfaction.

Moreover, the present results indicate that collective narcissism is associated with sensory processing sensitivity, a variable which pertains to a genetically based reactivity to environmental stimuli, especially negative, and increased depth of their cognitive processing (Aron and Aron, 1997). Highly sensitive people are more vulnerable to negative stimuli and to negative experiences undermining their psychological wellbeing (Belsky and Pluess, 2009; Booth et al., 2015). This suggests that dispositional negative emotionality may predispose people toward the collective narcissist belief about the in-group’s unrecognized importance. Such results suggest that collective narcissism may be underlined by dispositional deficits in emotional resilience and the ability to constructively self-soothe in face of adversity (Porges, 2007). Such interpretation is in line with Fromm’s (1973) and Adorno’s (1997) early claims that collective narcissism is a response to ‘ego fragility.’

However, heritability does not imply immutability. Negative emotional profile can be changed toward greater positive and prosocial emotionality (Fredrickson, 2001), especially when supported by appropriate interventions (e.g., Kok et al., 2013). Even if negative emotionality may predispose individuals toward collective narcissism, they may still access the benefits of positive social identity because collective narcissism overlaps with in-group satisfaction. Collective narcissists can access positive emotions and improve regulation of their negative emotions using the positive attitude toward their in-group. Positive emotionality not only signals, but also produces wellbeing. Positive and prosocial experiences boost emotional resilience. Positive (Oveis et al., 2009) and prosocial (Nelson et al., 2016; Stellar et al., 2017) emotionality is associated with greater life satisfaction. Indeed, the present results suggest that as long as collective narcissists are satisfied and proud members of their groups, they experience positive and self-transcendent emotions. Across three studies, the positive overlap with in-group satisfaction weakened the positive link between collective narcissism and negative emotionality, the association between collective narcissism and self-criticism, and the negative, unique relationship between collective narcissism and social connectedness, and gratitude. Collective narcissism was positively, indirectly linked to positive emotionality, compassion and self-compassion, as well as life satisfaction via in-group satisfaction.

It should be noted that the present results also indicate that as long as in-group satisfaction is related to collective narcissism, its relationship with gratitude and social connectedness is diminished. In addition, the overlap with collective narcissism links in-group satisfaction to negative emotions and self-criticism. Thus, the overlap with collective narcissism may be detrimental to people satisfied to be members of groups they deem valuable. Recent studies indicate that exposure to populist arguments, suggesting that the in-group’s greatness is threatened, increases collective narcissism (Marchlewska et al., 2018). This suggests that in social and political conditions that increase collective narcissism, people who highly value their group membership are likely to become unhappy. Thus, interventions and policies should focus on strengthening in-group satisfaction (which decreases collective narcissistic negativity), rather than collective narcissism (which decreases in-group satisfiers’ positivity).

### Limitations and Future Directions

The present studies offer an insight into distinct emotional profiles uniquely associated with collective narcissism and in-group satisfaction. Several limitations of the present studies should be mentioned. The studies are correlational and do not allow for firm conclusions regarding the directionality of examined relationships. Although it is likely that dispositional variables such as sensory processing sensitivity determine how people approach their in-groups, future studies would do well to examine these relationships in a longitudinal design. Such studies could, for example, examine indices of emotionality in time 1 and in-group attitudes in time 2. It is also possible that some of those relationships are reciprocal - for example an increase in positive emotions is expected to create further increase in time 2 (Fredrickson, 2001). Indeed, previous longitudinal studies indicated that personal control and high self-esteem lead to in-group satisfaction but in-group satisfaction also increases personal control and self-esteem (Cichocka et al., 2017; Golec de Zavala et al., 2019b).

Present studies used self-reported measures of emotionality. Future studies would do well examining the psychophysiological indices of emotionality such as the resting respiratory sinus arrhythmia indicative of individual vagal tone and functioning of the parasympathetic nervous system (Porges, 2007; Oveis et al., 2009). The present studies established the unique positive association of collective narcissism with the apparently innate trait of sensory processing sensitivity, supporting the proposition that collective narcissistic emotional negativity may be genetically based. This suggest that collective narcissism may be associated with low vagal tone which indicates functional deficits of the parasympathetic nervous (e.g., inability to self-soothe in face of adversity, Porges, 2007), or serotonin transmitter polymorphism (associated with greater responsiveness to negative stimuli, Acevedo et al., 2014; Homberg et al., 2016). Future studies examining whether collective narcissism and in-group satisfaction are associated with differences in functioning of the autonomous and central nervous system would be a valuable extension of the present investigation.

## Conclusion

This article argued that social identifications may not offer psychological resources for wellbeing when individuals hold a collective narcissist belief about their in-group. It was proposed, in line with the two factor theory of affect (Diener and Larsen, 1984; Emmons and Diener, 1985), that dispositional negative emotionality inclined people toward collective narcissism. Indeed, across four studies collective narcissism was positively related to negative emotionality and negatively related to social connectedness and gratitude, and it had no unique associations with positive emotionality and life satisfaction. Collective narcissism was also associated with the apparently innate trait of sensory processing sensitivity.

However, even when the positive evaluation of the in-group takes a form of collective narcissism, people can still access the psychological benefits that the group membership provides. Collective narcissism overlaps with in-group satisfaction, and via this overlap negative emotionality associated with collective narcissism can be gradually changed toward more positivity. The present manuscript presented an initial test of this proposition analyzing the indirect relationships between collective narcissism and positive and prosocial emotionality and life satisfaction, via in-group satisfaction. The empirical evidence indicated that in-group satisfaction was uniquely associated with positive and prosocial emotionality, lack of negative emotions and high life satisfaction. It suppressed the negative links between collective narcissism and negative emotionality. Collective narcissism was indirectly, positively linked to positive and prosocial emotionality and high life satisfaction via in-group satisfaction.

Such results indicate that different forms of positive attitude toward in-groups, labeled collective narcissism vs. in-group satisfaction, are related to different emotional profiles. This, at least partially, explains the etiology of the association of collective narcissism with hypersensitivity to intergroup threat and intergroup hostility. The positive overlap with in-group satisfaction offers a possibility of developing positive and prosocial emotionality among collective narcissists.

## Ethics Statement

The studies presented in this manuscript were carried out in accordance with the recommendations of ’the British Psychological Society with written informed consent from all participants, All participants gave informed consent in accordance with the Declaration of Helsinki. The studies were approved by the Ethics Committee at the Department of Psychology, Goldsmiths, University of London. Studies did not involve vulnerable populations. Participants consented to take part in the studies after reading the consent form.

## Author Contributions

AGdZ designed and conducted the studies and wrote up the manuscript.

## Conflict of Interest Statement

The author declares that the research was conducted in the absence of any commercial or financial relationships that could be construed as a potential conflict of interest.

## Footnotes

1 The concept of collective narcissism extends to the social level of the self; the concept of individual narcissism understood as a desire for continual external validation of an inflated self-view (e.g., Morf and Rhodewalt, 2001; Rhodewalt and Sorrow, 2003; Crocker and Park, 2004). It is assumed that as much as people can demand special recognition and privilege for themselves (as individual narcissists do), they can claim the same for the groups they belong to (as collective narcissists do). This does not mean that individual narcissists necessarily exaggerate their in-group’s importance. In fact, the evidence suggests this is not a very common case. However, the intergroup consequences of collective narcissism often parallel the interpersonal consequences of individual narcissism. Importantly, collective (but not individual) narcissism predicts intergroup behaviors and attitudes (for review and a more detailed discussion on the links between individual and collective narcissism, see Golec de Zavala et al., 2019a).

2 http://www.panelariadna.com

3 https://davidakenny.shinyapps.io/MedPower/

4 These data were initially collected to test a different hypothesis where only collective narcissism was a common variable. The study contained a failed experimental manipulation that did not affect the measures of emotionality.

5 This study was a part of a larger project in which a different hypothesis regarding collective narcissism was also examined. This hypothesis used different variables also measured in this study. The only overlapping variable is collective narcissism.
